# Nanospray Drying as a Novel Technique for the Manufacturing of Inhalable NSAID Powders

**DOI:** 10.1155/2014/838410

**Published:** 2014-12-16

**Authors:** Aquino Rita Patrizia, Stigliani Mariateresa, Del Gaudio Pasquale, Mencherini Teresa, Sansone Francesca, Russo Paola

**Affiliations:** Department of Pharmacy, University of Salerno, Via Giovanni Paolo II, No. 132, 84084 Fisciano, Italy

## Abstract

The aim of this research was to evaluate the potential of the nanospray drier as a novel apparatus for the manufacturing of a dry powder for inhalation containing ketoprofen lysinate, a nonsteroidal anti-inflammatory drug able to control the inflammation in cystic fibrosis patients. We produced several ketoprofen lysinate and leucine powder batches by means of nanospray dryer, studying the influence of process parameters on yield, particle properties (size distribution and morphology), and, mainly, aerodynamic properties of powders. Micronized particles were prepared from different hydroalcoholic solutions (alcohol content from 0 to 30% v/v) using ketoprofen in its lysine salt form and leucine as dispersibility enhancer in different ratios (from 5 to 15% w/w) with a total solid concentration ranging from 1 to 7% w/v. Results indicated that the spray head equipped with a 7 *µ*m nozzle produced powders too big to be inhaled. The reduction of nozzle size from 7 to 4 *µ*m led to smaller particles suitable for inhalation but, at the same time, caused a dramatic increase in process time. The selection of process variables, together with the nozzle pretreatment with a surfactant solution, allowed us to obtain a free flowing powder with satisfying aerosol performance, confirming the usefulness of the nanospray drier in the production of powder for inhalation.

## 1. Introduction

Spray drying is a one-step process widely used to obtain a powder from a solution, suspension, or emulsion, with the possibility of modulating powder physical and technological properties in relation to the specific use [1, 2]. In particular, in the field of formulations for inhalation, the appropriate tuning of process parameters may give the possibility to modify physical properties such as powder density, particle morphology, surface, and porosity, therefore dramatically influencing the aerodynamic performance for nasal and lung formulations [3–7]. Recently, an innovative spray dryer was developed, which claimed three unique patented technologies: a laminar airflow to decrease sample loss with minimal dead volume; a spray head system to produce small particles in a very narrow size distribution; and an electrostatic particle collector to obtain high yields and recover even the smallest particles [8–10].

Different from standard spray drying apparatus characterized by a pneumatic nozzle, in this case liquid feed droplets are generated by a piezoelectric system, vibrating a thin, stainless steel membrane. The membrane features a series of precise micron-sized holes (spray meshes of 4.0, 5.5, or 7.0 *μ*m hole size): after an ultrasonic frequency (60 kHz), membrane vibrates, ejecting precisely sized droplets at high speed. Moreover, the dried solid particles are electrostatically charged and collected in an electrostatic collector, so that their separation seems to be independent of particle mass as in standard cyclones and to allow collection of smaller particles; consequently, process yield may be improved [11, 12].

Among drug formulations, powders for inhalation are certainly those whose efficacy depends mainly on the particle size, strongly influencing the aerosol deposition [13, 14]. Thus, the possibility to reduce and control particle diameter compared to standard spray drying system makes the nanospray drying a promising technology for the production of inhalation powders, as confirmed by recent studies [15, 16].

The aim of this research was to evaluate the potential of this innovative technology for the manufacturing of a dry powder for inhalation containing ketoprofen lysinate (Klys) as model drug for inflammation control in cystic fibrosis (CF) patients. Respirable engineered particles of this NSAID were previously prepared by cospray drying the active pharmaceutical ingredient (API) and leucine (leu) as safe excipient. The use of amino acid as a powder dispersibility enhancer showed no influence both on drug dissolution and permeation and on viability of ΔF 508 CF (CuFi1) cells [17]. With this aim we produced several ketoprofen lysinate and leucine powder batches by nanospray dryer, studying the influence of process parameters on yield, particle properties (size distribution and morphology), and, mainly, aerodynamic properties of the resulting powders.

## 2. Materials and Methods

### 2.1. Materials

Ketoprofen lysine salt was kindly donated by Dompè SpA (L'Aquila, Italy); L-leucine was supplied by Sigma Aldrich (Milan, Italy). Isopropyl alcohol (IPA) (for analysis, USP grade) was purchased from Carlo Erba Reagents (Milan, Italy). Other solvents and chemicals were of analytical grade. Size 3 gelatine capsules were purchased from Dermolife (Trento, Italy). The device used for aerodynamic tests was the* monodose DPI model 7* kindly donated by Plastiape SpA (Milan, Italy).

### 2.2. Methods

#### 2.2.1. Powder Preparation

Micronized particles were prepared from different hydroalcoholic solutions (IPA from 0 to 30% v/v) containing ketoprofen in its lysinate salt form and leucine as dispersibility enhancer in different ratios (from 5 to 15% w/w) with a total solid concentration ranging from 1 to 7% w/v. Besides solutions compositions, the operating conditions of nanospray drier were tuned in order to study their effect on powder technological properties. In detail, inlet temperature ranged between 60 and 110°C, while air flow rate (100 L/min), feed rate (1.5 mL/min), and relative spray rate (100%) were kept constant. Solutions were sprayed alternatively using nozzles with mesh diameters of 4.0, 5.5, and 7.0 *μ*m. A nozzle pretreatment with surfactant solutions was also carried out, aiming to reduce the processing time.

#### 2.2.2. Ketoprofen Lysinate Quantification

Ketoprofen lysinate was quantified by UV detection (Evolution 201, Thermo Fisher Scientific, Spectral, Ozzano dell'Emilia, Bologna, Italy) at a wavelength of 259 nm [18], using 1 cm SUPRASIL quartz cell (Hellma 100-QS, HELLMA Italia Srl, Milan, Italy). The analytic method was validated using standard solutions of ketoprofen lysinate in the range of 5–30 *μ*g/mL (*y* = 0.0407*x* + 0.0048; *R* ^2^ = 0.9998).

#### 2.2.3. Particle Size

Particle size of both raw materials and engineered particles was determined using a light-scattering laser granulometer equipped with a tornado powder dispersing system [17] (LS 13 320 Beckman Coulter Inc., FL, USA). The LS 13 320 uses a 5 mW laser diode with a wavelength of 750 nm and reverse Fourier optics incorporated in a fibre optic spatial filter and binocular lens systems. The particle size was obtained by specific software using Mie theory to produce an optimal analysis of the light energy distribution. The tornado module leads to a dispersion similar to the one achieved when the samples are run wet, without using any solvent which can alter powder surface properties. Samples were charged into a plastic cylinder in order to obtain an obscuration value between 4 and 8%.

Results were expressed as *d*_50_ and span, defined as [*d*(90) − *d*(10)]/*d*(50), where *d*(10), *d*(50), and *d*(90) indicate diameters at the 10th, 50th, and 90th percentiles of the particle size distribution, respectively.

#### 2.2.4. Scanning Electron Microscopy (SEM)

Morphology of raw materials and engineered particles was investigated using a scanning electron microscope (SEM) Zeiss EVO MA10 (Carl Zeiss SMT AG, München-Hallbergmoos, Germany) operating at 14 kV [19].

### 2.3. Aerodynamic Behaviour Evaluation

Powders aerodynamic properties were assessed by Andersen cascade impactor (apparatus D, Eur. Ph. 6.0, ACI, Westech Instrument Services Ltd., Bedfordshire, UK), adjusted for use at a flow rate of 60 L/min as described elsewhere [3]. The device used to aerosolize the powders was the* monodose*, a breath-activated, reusable dry powder inhaler (DPI), working with a size 3 capsule. The capsule is horizontally inserted into the pulverization chamber and pierced by two needles at the bottom and at upper side: the inhaled air creates a turbulence that shakes and twists the capsule, facilitating its emptying. The ACI was assembled placing a filter paper on the filter stage and the* monodose* DPI was fitted into a rubber mouth piece attached to the metal throat.

Four hard gelatine capsules (size 3) were filled manually with 40 ± 0.5 mg of sample. Each capsule was introduced into the* monodose* DPI and pierced. The vacuum pump was actuated for 4 s. The powder deposited into the different stages was recovered by plunging each plate and the stage below into distilled water (5–500 mL depending on the stage number). Drug content was assessed by UV measurements. The emitted dose was gravimetrically determined and expressed as percentage of powder exiting the device versus the amount of powder introduced into the capsule. The cumulative mass of powder with a diameter lower than the stated size of each stage was calculated and plotted as a percentage of recovered powder* versus* cut-off diameter. The mass median aerodynamic diameter (MMAD) of the particles was extrapolated from the graph, according to the Eur. Ph. 6.0. From the same plot, the fine particle dose (FPD), that is, the mass of drug with a particle size less than 5 *μ*m, and the fine particle fraction (FPF), that is, the fraction of drug emitted from the device with a particle size less than 5 *μ*m, were determined.* In vitro* deposition experiments were performed on three batches with three replicates each [20].

## 3. Results and Discussion

### 3.1. Manufacturing and Characterization of Powders

Several ketoprofen lysinate and leucine powders were produced by nanospray drier, with the aim of evaluating the effect of different operative conditions on physicochemical properties and aerodynamic performance of microparticles produced. On the basis of our previous research [17] in the first series of experiments Klys/leu ratio and composition of the hydroalcoholic feed were set at 85/15 w/w and 70/30 v/v water/IPA, respectively. Powder batches and main characteristics are summarized in Table 1.

The first process variable considered was the dimension of spray head which at the beginning was set at 7 *μ*m, as to nozzle diameter (batch #1 and #2). Both liquid feeds containing a powder/solid concentration from 3 to 5% w/v led to free flowing powders with a very high yield (>80%); however dimensional analysis showed particles in a dimensional range expected to be not suitable for inhalation (*d*_50_ > 6 *μ*m). Therefore, with the aim of obtaining particles with a smaller geometric diameter, the 4 *μ*m nozzle was chosen, exploring the effect of both temperature and total solid concentration in the feed on powder properties (Table 1). Generally, using a 4 *μ*m nozzle, lower powder concentration (1–3% w/v, batch #4, #6, and #7) in the liquid feed led to a sticky product, difficult to handle and to test as to particle size and aerodynamic performance. Accordingly, for these powders, process yields were not satisfactory. More dense feeds, containing higher solute concentration (5% w/v of ketoprofen lysinate and leucine, batch #3 and #5), improved process yield, producing powder in a good dimensional range (*d*_50_ 2.4–3.2 *μ*m). Moreover, it is notable that nanospray allows the considerable reduction of the inlet temperature from 110°C up to a value of 70°C, in comparison to standard spray drying technology, and this appears as a very important result for the processability of thermolabile active compounds.

**Figure 1:**
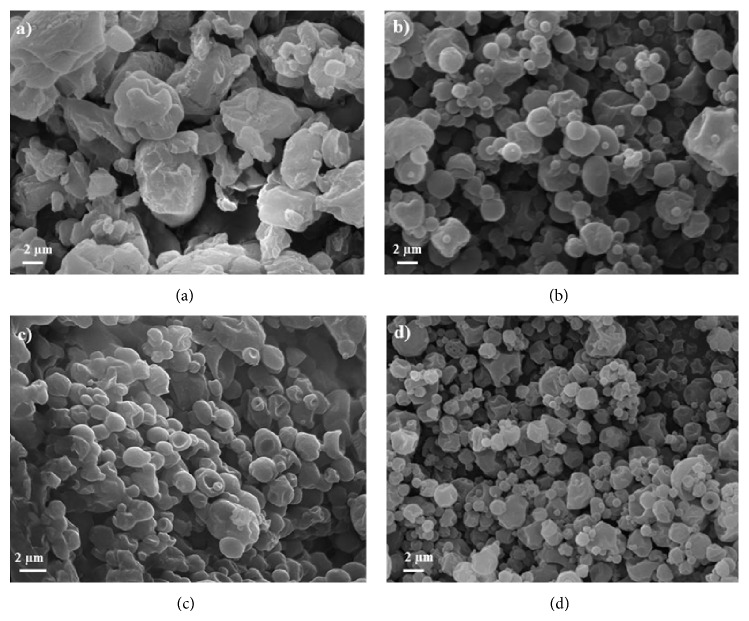
SEM pictures of powders obtained from (a) #2 (110°C, 3% w/v, 7 *μ*m nozzle), (b) #1 (110°C, 5% w/v, 7 *μ*m nozzle), (c) #4 (110°C, 3% w/v, 4 *μ*m nozzle), and (d) #3 (110°C, 5% w/v, 4 *μ*m nozzle).

Morphology study evidenced that particles were irregularly shaped but well separated from each other (Figures 1(a) and 1(b)) when produced by means of a 7 *μ*m nozzle (#2, #1). Powders obtained using a 4 *μ*m nozzle were sensibly smaller in diameter as clearly visible in SEM picture (Figures 1(c) and 1(d)), but only the highest feed concentration (5% w/v) led to particles not agglomerated (Figure 1(d), #3), as predictable from very low process yield and powder stickiness.

*In vitro* deposition behaviour of nonsticky powders was evaluated using the* monodose DPI model 7* as device for the dose erogation and the Andersen cascade as impactor. The emitted dose for all nanospray-dried batches was almost 100% of the charged formulation, indicating a very efficient deaggregation of powders by means of the selected device. The dose recovered from the impactor was higher than 80% in all cases (data not shown). As expected from powders geometric diameter (>6 *μ*m), (Table 1) batches produced by means of a 7 *μ*m nozzle showed poor aerodynamic properties, with FPF <30%.

**Figure 2:**
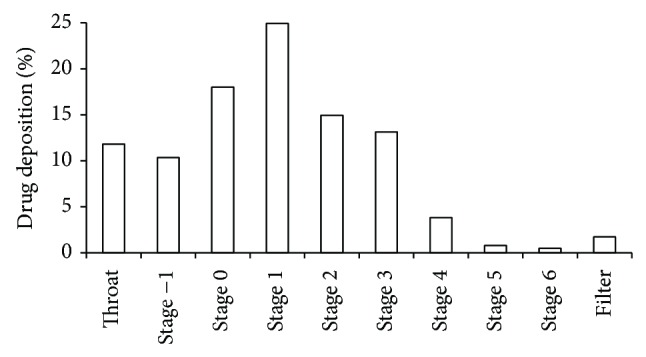
Andersen cascade impactor deposition pattern after the aerosolization of powder (batch #5) sprayed with a 4 *μ*m nozzle at 70°C (#5%* N*_*4*_ *T*_*70*_).

Among powders produced by means of the 4 *μ*m nozzle, very interesting aerodynamic properties were evidenced for batch #5 showing FPF of 66.3%, together with the smallest geometric diameter and a high process yield (5%* N*_*4*_ *T*_*70*_, Table 2).  Figure 2 shows the amount of drug deposition on the throat, on stages 1 to 7, and on the filter, expressed as percentages of the total powder recovered from the impactor. Confirming its excellent aerodynamic performance, batch #5 was characterized by a very low deposition on the throat and onto the upper stages of the Andersen cascade impactor (Figure 2).

**Figure 3:**
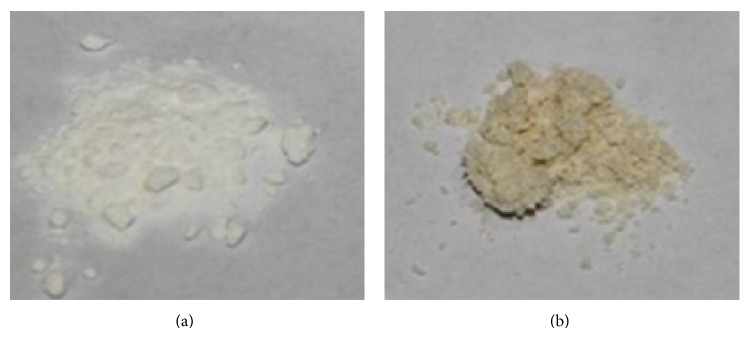
Picture of Klys/leu powders after nanospray process using 7 (#1, a) and 4 *μ*m nozzle (#5, b).

However, in addition to an improvement of the aerodynamic properties of the powders, the reduction of nozzle size from 7 to 4 *μ*m led also to a dramatic increase in process time (Table 1), moving from 1.11 to 0.06 mL/min of liquid feed processed. The portion of liquid pumped into the atomization head and not sprayed went back to the feed container in a continuous loop, exposing the sample to the inlet temperature for very long times. In the case of Klys, this effect caused the production of yellowish powders (Figure 3).

Several attempts were pursued with the purpose of reducing thermal stress of the processing fluid (i.e., use of ice bath and reduction of batch volumes), with unsatisfactory results. Finally, with the aim of reducing viscosity at the nozzle and increasing droplet formation efficiency, nozzle was dressed with a film of surfactant dipping the vibrating membrane into a span 80 n-hexane solution (0.05% w/v) and allowing the solvent to evaporate. As expected, the thin film of surfactant changed the influence of process parameters on powder properties, so that a new setup of the apparatus was necessary. The main process variables considered were total powder concentration (ranging from 5 to 7% w/v), drug/leu ratio (from 85/15 to 90/10 w/w), and water content (from 70 to 100% v/v) in the hydroalcoholic feeds. The characteristics of the main batches prepared with the surfactant treated nozzle are summarized in Table 3.

The surfactant layer on the spray head membrane generally caused speeding-up of the atomization (up to 0.42 mL/min) and a consequent reduction in process time.

**Figure 4:**
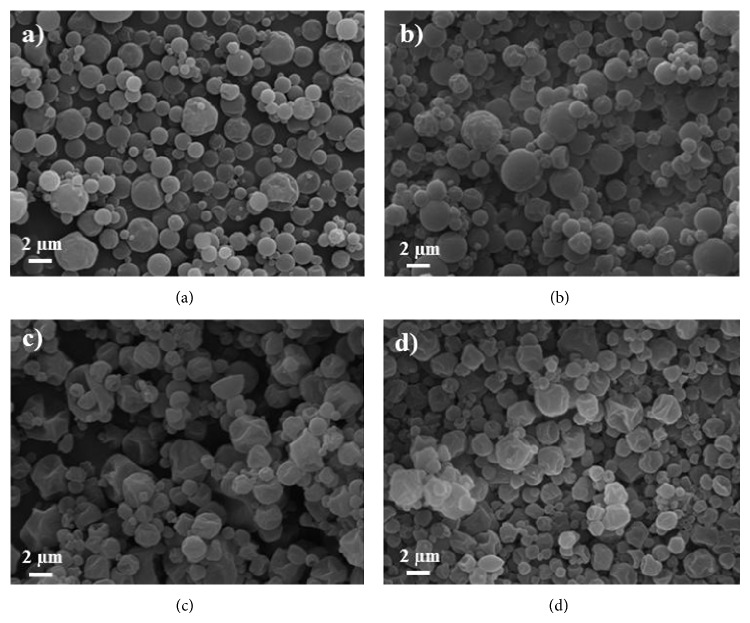
SEM pictures of powders obtained from (a) #14 (6% w/v, water/IPA 100/0 v/v, 10% leu), (b) #13 (6% w/v, water/IPA 90/10 v/v, 10% leu), (c) #10 (6% w/v, water/IPA 100/10 v/v, 15% leu), and (d) #11 (6% w/v, water/IPA 90/10 v/v, 15% leu).

As evidenced by SEM pictures reported in Figure 4, the amount of leucine influenced particle morphology and surface properties, for both aqueous and hydroalcoholic liquid feeds processed. In particular, particles from powders containing 10% leucine were spherical in shape (Figures 4(a) and 4(b)), while particles from powders containing higher amount of leucine (15%, Figures 4(c) and 4(d)) were wrinkled and corrugated. This is a very important effect, since an increase in particle surface roughness corresponds to an increase in shape factor and a consequent reduction in the aerodynamic diameter.

Finally, concerning powder technological properties, improvements were obtained by increasing the concentration of dry content up to 6% and/or reducing the amount of organic solvent in the liquid feeds (from 30 to 0% v/v), resulting, in both cases, in an increase of solution viscosity. In particular, batches 11 and 13 produced from 90/10 v/v hydroalcoholic solutions were white and free-flowing powders: among these, batch 11 containing 15% w/w of leucine represented the most promising product, in terms of process efficiency (0.42 mL/min of liquid feed processed and 75.3% yield).

Besides process efficiency, DPI containing #11 presented also the highest fraction of drug with an aerodynamic diameter less than 5 *μ*m, as reported in Table 4.

Increasing particle roughness and corrugation, leucine positively influenced powders aerodynamic performance, with FPF values up to 60.9%.

## 4. Conclusions

The nanospray drier system seems to be an efficient alternative to standard spray drying in formulating dry powders with reduced geometric diameter and increased aerosol performance. Certainly, the accurate tuning of process variables is necessary to allow the preparation of fine powders with physicochemical and aerodynamic properties suitable for inhalation. In the case of ketoprofen lysinate, using the smallest nozzle (4 *μ*m) process times were considerably high, resulting in brownish powders. To reduce the process time and gain a good yield, a surfactant thin film covering the nozzle was required, with the aim of increasing drug solution passage through the micron-sized holes of the membrane, accelerating powder production. The selection of process variables allowed us to obtain a white powder with satisfying aerosol performance and be able to release 18 mg of fine particles after one actuation of the* monodose* device.

## Figures and Tables

**Table 1:** Klys/leu powders produced by nanospray drier: process parameters, yield, drug content, and particle size distribution (15% leu and 30% IPA).

Batch	*T*_inlet_ (°C)	Total powder concentration (% w/v)	Nozzle (*µ*m)	Yield (%)	*D*_50_ (*µ*m) and span	Drug content (% p/p)	Process time (mL/min)
#1	110	5%	7	87.0 ± 2.8	6.6 (1.7)	79.4 ± 1.5	1.11
#2	110	3%	7	82.3 ± 3.2	6.2 (1.7)	77.0 ± 2.1	1.11
#3	110	5%	4	62.3 ± 2.8	3.2 (1.7)	82.9 ± 0.8	0.13
#4	110	3%	4	43.0 ± 4.2	—^a^	79.2 ± 3.1	0.10
#5	70	5%	4	77.6 ± 1.6	2.4 (1.7)	75.3 ± 0.4	0.06
#6	70	3%	4	56.7 ± 1.0	—^a^	78.5 ± 1.1	0.06
#7	70	1%	4	52.1 ± 1.8	—^a^	80.1 ± 1.3	0.08
#8	60	5%	4	27.9 ± 7.1	—^a^	73.5 ± 1.2	0.04
#9	70	5%	5.5	68.6 ± 1.9	3.1 (2.4)	72.4 ± 0.6	0.33

^a^Powder stickiness prevented measure.

**Table 2:** Aerodynamic properties of nanospray-dried Klys/leu (15% leu, 30% IPA) powders after ACI experiments: emitted dose (ED), mass median aerodynamic diameter (MMAD), fine particles fraction (FPF), and fine particle dose (FPD).

Batch	Feed concentration (%), nozzle (*N*), and temperature (*T*)	Emitted dose (%)	MMAD (*µ*m)	FPF (%)	FPD (mg)
#1	5% *N*_7_ *T*_110_	99.2 ± 0.1	5.69 ± 0.23	21.9 ± 2.5	6.27 ± 0.5
#2	3% *N*_7_ *T*_110_	99.2 ± 0.1	5.44 ± 0.55	29.5 ± 2.3	8.16 ± 0.24
#3	5% *N*_4_ *T*_110_	99.1 ± 0.1	4.25 ± 0.12	50.4 ± 1.9	12.7 ± 1.1
#5	5% *N*_4_ *T*_70_	100.0 ± 0.1	3.72 ± 0.07	66.3 ± 1.0	16.9 ± 0.6
#6	3% *N*_4_ *T*_70_	96.7 ± 4.2	6.25 ± 0.11	31.6 ± 1.6	7.85 ± 1.2
#9	5% *N*_5.5_ *T*_70_	97.3 ± 1.0	4.58 ± 0.14	45.6 ± 2.2	12.2 ± 1.3

**Table 3:** Process parameters and physical characteristics of Klys/leu particles sprayed through a surfactant treated nozzle (70°C, 4 *µ*m nozzle): liquid feed composition, yield, and dimensional distribution.

Batch	Total powder concentration (% w/v)	Leu concentration (% w/w)	Water/IPA (% v/v)	Yield (%)	*D*_50_ (*µ*m) and span	Process time (mL/min)
#5a	5%	15%	70/30	33.1 ± 9.2	—^a^	0.21
#5b	6%	15%	70/30	45.1 ± 3.5	—^a^	0.21
#5c	5%	10%	70/30	74.8 ± 2.5	—^a^	0.21
#5d	6%	10%	70/30	75.2 ± 1.0	3.01 (1.80)	0.21
#5e	7%	10%	70/30	18.2 ± 7.5	—^a^	0.21
#10	6%	15%	100/0	71.7 ± 1.2	3.14 (1.86)	0.14
#11	6%	15%	90/10	75.3 ± 0.5	2.84 (1.63)	0.42
#12	6%	15%	80/20	68.7 ± 2.6	2.94 (1.90)	0.06
#13	6%	10%	90/10	76.2 ± 3.1	2.52 (1.90)	0.21
#14	6%	10%	100/0	65.5 ± 1.5	3.14 (1.69)	0.10

^a^Powder stickiness prevented measure.

**Table 4:** Aerodynamic properties of Klys/leu powders sprayed through a surfactant treated nozzle (70°C, 4 *µ*m nozzle, and 6% w/v feed) after ACI experiments: emitted dose (ED), mass median aerodynamic diameter (MMAD), fine particles fraction (FPF), and fine particle dose (FPD).

Batch	Leu concentration (% w/w)	Water/IPA (% v/v)	Emitted dose (%)	MMAD (*µ*m)	FPF (%)	FPD (mg)
#5d	10%	70/30	98.4 ± 2.9	4.25 ± 0.12	54.4 ± 0.3	15.8 ± 0.4
#10	15%	100/0	100.1 ± 0.1	4.02 ± 0.05	54.8 ± 1.1	18.2 ± 0.7
#11	15%	90/10	99.7 ± 0.3	3.83 ± 0.02	60.9 ± 1.2	18.0 ± 0.4
#12	15%	80/20	99.9 ± 0.2	4.19 ± 0.20	55.3 ± 2.5	16.6 ± 1.1
#13	10%	90/10	100.8 ± 0.1	4.31 ± 0.22	49.9 ± 3.2	15.5 ± 0.9
